# 189. Optimizing Empiric Antibiotic Therapy: a Probabilistic Approach

**DOI:** 10.1093/ofid/ofac492.267

**Published:** 2022-12-15

**Authors:** Bráulio R G M Couto, Adelino M Freire Júnior, Mozar Castro Neto, Carolina Rodrigues, Mariana Melo, Edna M M Leite, Simony Gonçalves, Virginia Andrade, Lívia Miranda, André Couto, Jeruza Romaniello, Emerson Braga, Estevão Urbano, Herbert Fernandes, Carlos E Starling

**Affiliations:** AMECI – Associação Mineira de Epidemiologia e Controle de Infecções, Belo Horizonte, Minas Gerais, Brazil; Hospital Felício Rocho, Belo Horizonte, Minas Gerais, Brazil; Hospital Felício Rocho, Belo Horizonte, Minas Gerais, Brazil; Hospital Felício Rocho, Belo Horizonte, Minas Gerais, Brazil; Hospital Metropolitana Doutor Célio de Castro, Belo Horizonte, Minas Gerais, Brazil; Hospital Risoleta Tolentino Neves, Belo Horizonte, Minas Gerais, Brazil; Hospital Risoleta Tolentino Neves, Belo Horizonte, Minas Gerais, Brazil; Hospital Madre Teresa, Belo Horizonte, Minas Gerais, Brazil; Faculdade Dinâmica Vale do Piranga - FADIP, Viçosa, Minas Gerais, Brazil; Faculdade Ciências Médicas de Minas Gerais, Belo Horizonte, Minas Gerais, Brazil; Hospital Evangélico, Belo Horizonte, Minas Gerais, Brazil; Hospital Municipal Odilon Behrens, Belo Horizonte, Minas Gerais, Brazil; Biocor Instituto, Belo Horizonte, Minas Gerais, Brazil; Hospital Ibiapaba, Barbacena, Minas Gerais, Brazil; Sociedade Mineira de Infectologia - SMI, Belo Horizonte, Minas Gerais, Brazil

## Abstract

**Background:**

How to start optimal antibiotic therapy before the results of cultures and antimicrobial susceptibility tests are available? Here, we use the law of total probability to present a probabilistic approach based on antibiograms of bacterial isolates from healthcare and community-acquired infections to optimizing empiric antibiotic therapy.

**Methods:**

Data on the microbiology of healthcare and community-acquired infections were analyzed from hospitals in Belo Horizonte, a three million inhabitants city from Brazil. Healthcare infections were defined by the National Healthcare Safety Network (NHSN)/CDC protocols. Only data obtained from infections with positive culture, both hospital and community, were considered. The success rate of an antibiotic (ATB) regimen, considering just one drug individually (monotherapy), was calculated by Law of Total Probability (Fig 1). In this sense, if a microorganism has not been tested for a specific antimicrobial, then, by definition, it was considered an antibiotic failure. For a regimen with more than one antibiotic, if the microorganism is sensitive to one of them, then it was considered a success of the scheme. For calculating the success probability of two or three antimicrobials A, B, and C, simultaneously (Fig 2), i.e., P(A and B) or P(A and B and C), the sensitivity to an antimicrobial was considered independent of sensitivity to any other. Then, P(A and B) = P(A) * P(B), and P(A and B and C) = P(A)*P(B) *P(C).

**Figure 1:**
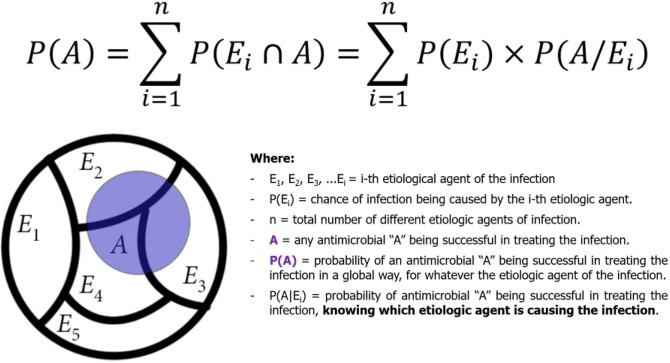
– Law of total probability: success rate of an antibiotic considering just one drug individually (monotherapy).

**Figure 2:**
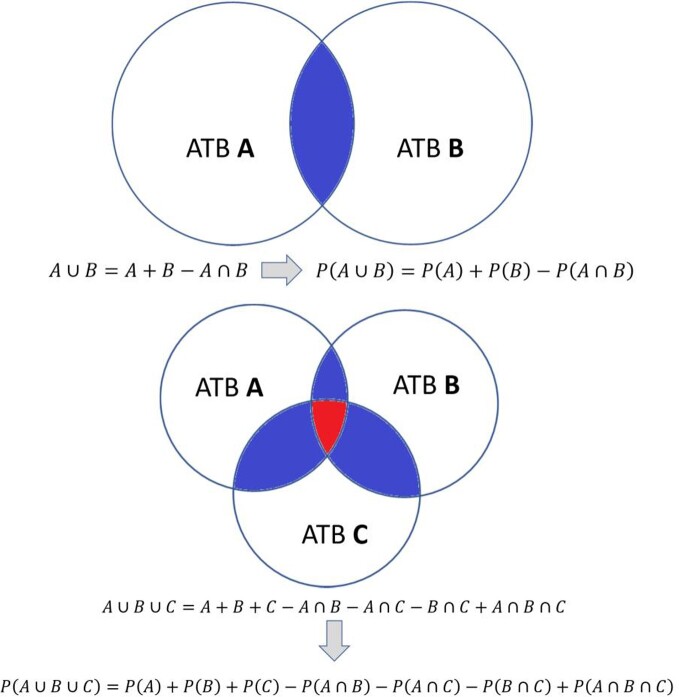
– Probability of the union of two events, success of ATB A or ATB B, and union of three events, success of ATB A or ATB B or ATB C.

**Results:**

Microbiologic data from hospital acquired infections (HAI) and community-acquired infections (CAI) are analyzed once a year. Empiric antibiotic therapy to HAI were proposed for urinary tract infections (UTI), bloodstream infections (BSI), and pneumonia (Figures 2 and 3). Empiric antibiotic therapy to community-acquired infections were developed for UTI, pneumonia, gastrointestinal system infection, bone and joint infection, and skin and soft tissue infection.

**Figure 3:**
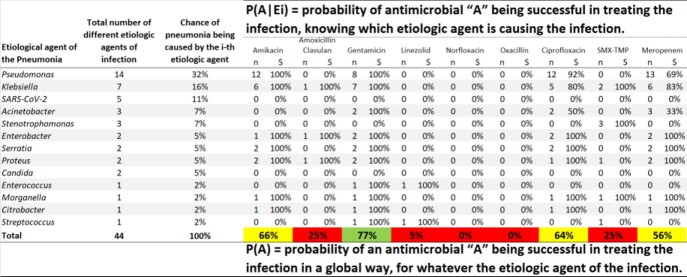
– Success rate of each antibiotic alone, considering just one drug individually (monotherapy): analysis of hospital-acquired pneumonia.

**Figure 4:**
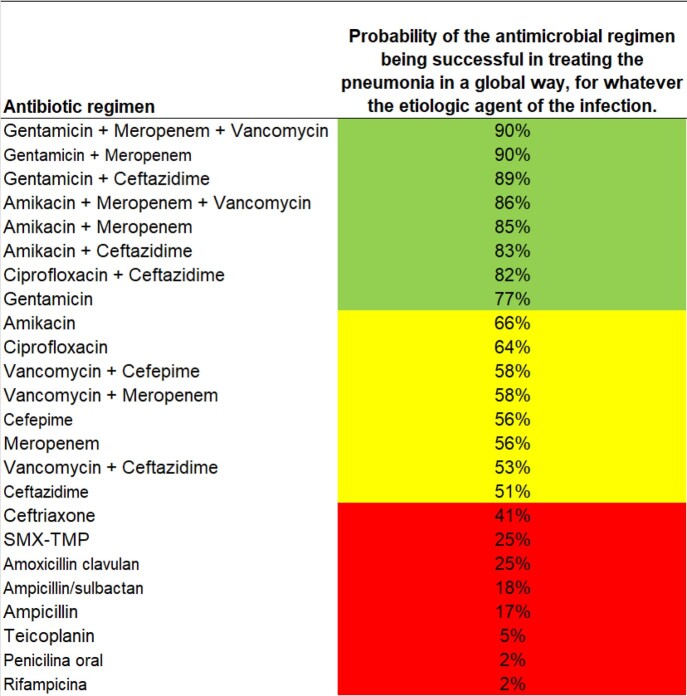
– Success rate of one, two or three antibiotics: analysis of hospital-acquired pneumonia.

**Fig 5:**
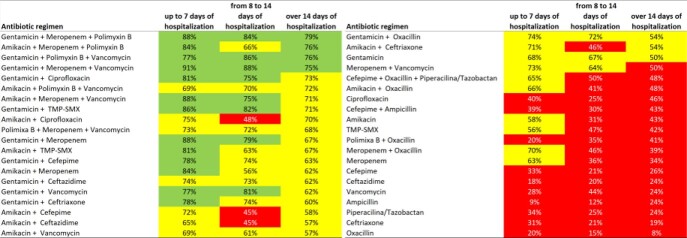
- Probability of the antimicrobial regimen being successful in treating an infection according to the length of stay at hospital.

**Conclusion:**

We presented here a probabilistic approach to empiric antibiotic therapy. The next step is to validate all proposed regimens, that can be used to improve the success likelihood of empiric antibiotic decision making.

**Disclosures:**

**All Authors**: No reported disclosures.

